# Fe^3+^-Modulated In Situ Formation of Hydrogels with Tunable Mechanical Properties

**DOI:** 10.3390/gels11080586

**Published:** 2025-07-30

**Authors:** Lihan Rong, Tianqi Guan, Xinyi Fan, Wenjie Zhi, Rui Zhou, Feng Li, Yuyan Liu

**Affiliations:** 1 College of Physics and Electronic Information Engineering, Neijiang Normal University, Neijiang 641112, China; 17828856639@163.com (T.G.); 18882615119@163.com (X.F.); 19934246087@163.com (W.Z.); 18283264132@163.com (R.Z.); 13981408134@163.com (F.L.); aa3037465663@outlook.com (Y.L.); 2 Neijiang Optoelectronic Devices Engineering Research Center, Neijiang 641112, China

**Keywords:** tunable mechanical properties, Fe^3+^ for hydrogels modulation, PET-RAFT polymerization, strain sensors

## Abstract

Fe^3+^-incorporated hydrogels are particularly valuable for wearable devices due to their tunable mechanical properties and ionic conductivity. However, conventional immersion-based fabrication fundamentally limits hydrogel performance because of heterogeneous ion distribution, ionic leaching, and scalability limitations. To overcome these challenges, we report a novel one-pot strategy where controlled amounts of Fe^3+^ are directly added to polyacrylamide-sodium acrylate (PAM-SA) precursor solutions, ensuring homogeneous ion distribution. Combining this with Photoinduced Electron/Energy Transfer Reversible Addition–Fragmentation Chain Transfer (PET-RAFT) polymerization enables efficient hydrogel fabrication under open-vessel conditions, improving its scalability. Fe^3+^ concentration achieves unprecedented modulation of mechanical properties: Young’s modulus (10 to 150 kPa), toughness (0.26 to 2.3 MJ/m^3^), and strain at break (800% to 2500%). The hydrogels also exhibit excellent compressibility (90% strain recovery), energy dissipation (>90% dissipation efficiency at optimal Fe^3+^ levels), and universal adhesion to diverse surfaces (plastic, metal, PTFE, and cardboard). Finally, these Fe^3+^-incorporated hydrogels demonstrated high effectiveness as strain sensors for monitoring finger/elbow movements, with gauge factors dependent on composition. This work provides a scalable, oxygen-tolerant route to tunable hydrogels for advanced wearable devices.

## 1. Introduction

Wearable devices have garnered significant interest due to their inherent portability and functional versatility [1,2,3]. Hydrogels, which are soft materials composed of three-dimensional polymer networks with high water content [4,5,6], have emerged as particularly promising candidates for fabricating key components such as strain sensors [7,8,9], body fluid detectors [10,11,12], and physiological signal monitors [6,13,14,15]. To enhance the sensitivity and reliability of these hydrogel-based devices, conductive metal ions are incorporated to improve mechanical robustness and ionic conductivity [16,17,18]. Among them, ferric ions (Fe^3+^) are particularly notable for their strong chelation capability with functional groups like carboxylates and catechols, a property extensively leveraged in the creation of high-performance hydrogels [19,20,21]. For instance, Ajoy et al. [22] prepared a Fe^3+^-rich lignin hydrogel for supercapacitor and sensor applications, where the chelation between catechol groups and Fe^3+^ largely improved the mechanical properties and conductivity. Sun et al. [23] reported a dual network hydrogel with polyaniline (PANI) and poly(acrylamie-co-acrylic acid) (P(AM-co-AA)). In this system, the coordination of Fe3+ and -COOH reinforced the hydrogel, forming a high-performance material for human motion strain sensors.

However, conventional methods for introducing Fe^3+^ into hydrogels primarily rely on post-synthesis immersion in high-concentration Fe^3+^ solutions. This approach presents several key challenges for achieving optimal hydrogel performance: First, soaking induces the rapid formation of a dense outer layer due to preferential surface contact and reaction, severely inhibiting Fe^3+^ diffusion into the hydrogel network center. This heterogeneous distribution compromises mechanical integrity and ionic conductivity throughout the material [24]. Second, the immersion process inherently lacks precise control over the incorporated Fe^3+^ amount, invariably leading to excessive free ions. This surplus intensifies the Fe^3+^/Fe^2+^ redox cycle, impairing essential coordination bonds and weakening the mechanical properties [25]. Third, the aqueous immersion step imposes a fundamental barrier to scalability for mass production [26]. This limitation persists because industrial-scale synthesis relies predominantly on stirred tank reactors—the standard equipment for both academic and commercial hydrogel production [5]. Immersing bulk hydrogels in these systems remains technologically challenging due to container volume constraints and difficulties in maintaining uniform ion distribution in industrial-scale batches.

To overcome these limitations, we developed a novel strategy involving the direct addition of a predetermined amount of Fe^3+^ to the precursor solution. By integrating this with Photoinduced Electron/Energy Transfer-Reversible Addition–Fragmentation Chain Transfer (PET-RAFT) polymerization, we successfully fabricated hydrogels under open-vessel conditions, even with Fe^3+^ presence. Applied here to polyacrylamide-sodium acrylate (PAM-SA) hydrogels, this method demonstrated exceptional controllability of mechanical properties. Mechanical properties, such as strength and Young’s modulus, can be readily tuned by simply adjusting the initial monomer ratios and Fe^3+^ concentrations within the precursor solution. These tunable high-performance polyelectrolyte hydrogels exhibit significant potential for application in advanced flexible strain and pressure sensors, paving the way for next-generation wearable devices.

## 2. Results and Discussion

### 2.1. Hydrogel Preparation

**Figure 1:**
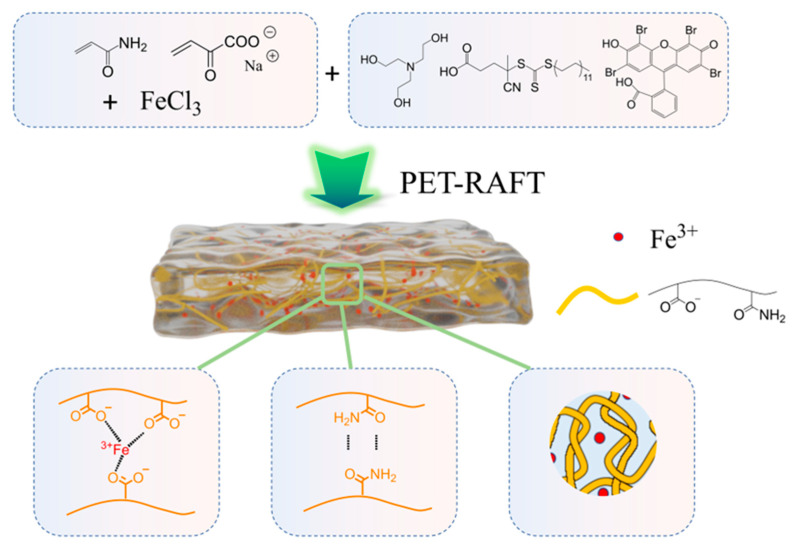
Schematic illustration of the preparation of PxMyFez samples via PET-RAFT polymerization and their interaction, including Fe^3+^ chelation, hydrogen bonding and physical entanglements.

Hydrogels were prepared using a one-pot method at room temperature under open-vessel conditions. The desired amount of monomers (acrylamide (AM) and sodium acrylate (SA)), Eosin Y (EY), Triethanolamine (TEOH), 4-Cyano-4-(((dodecylthio)carbonothioyl)thio)pentanoic acid (CDTPA), N,N’-Methylenebisacrylamide (MBAA, crosslinker) and FeCl_3_ were dissolved in deionized (DI) water. Then the mixture was transferred and irradiated by green LED light under open-vessel conditions for 1 h, as shown in Figure 1. Three series of samples were prepared to investigate the influence of SA amount, crosslinker amount, and Fe^3+^ amount on the mechanical properties. These hydrogels were named as PxMyFez, where x represents the mass ratio of two monomers of AM:SA, y denotes the final amount of MBAA, and z indicates the final amount of FeCl_3_. Detailed compositions are shown in Table 1.

To prove the PET-RAFT process occurred, we performed a control experiment in a dark environment (Figure S1). Precursor solutions of P1M0.5Fe0~P1M0.5Fe3 were transferred to a polytetrafluoroethylene (PTFE) mold and placed in a dark chamber. After 1 h in the dark environment, the P1M0.5Fe0 sample remained liquid, whereas P1M0.5Fe1 and P1M0.5Fe2 underwent gelation due to the interaction between the SA monomer and Fe^3+^. Interestingly, P1M0.5Fe3 formed a sticky liquid rather than a gel, possibly due to the uneven distribution and non-uniform chelation of excess Fe^3+^ ions. Subsequent 1 h irradiation cured all samples, confirming successful polymerization (Figure S1). The observed color change was attributed to the decomposition of EY. The hydrogel samples experienced a rapid gelation upon FeCl_3_ addition because of the chelation between Fe^3+^ and COO^−^. Normally, the gelation occurred in 5 min. Therefore, the synthesis of the hydrogel required a fast transition to the PTFE mold after the mixing. The interaction between the Fe^3+^ ions and COO^−^ groups was further confirmed with the Fourier-Transform Infrared (FTIR) spectra. A new peak appearing at 1652 cm^−1^ represented the characteristic peak of the carbonyl (C=O) coordinated with Fe^3+^, directly evidencing the successful chelation (Figure S2).

### 2.2. Tensile Tests

**Figure 2:**
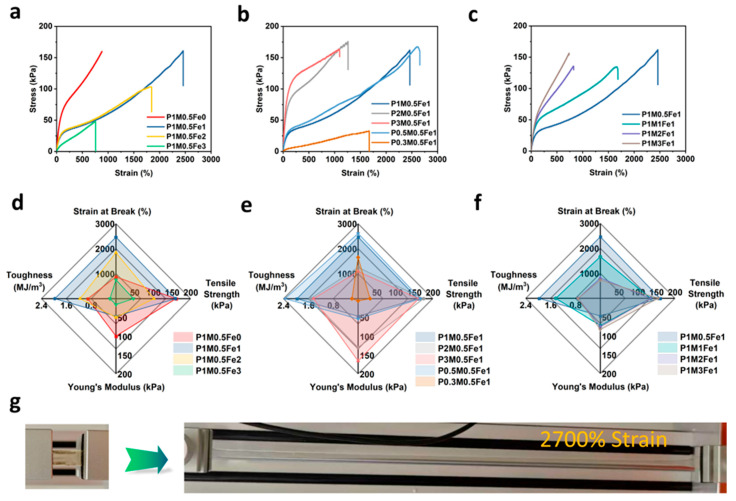
(**a**) Tensile stress–strain curves of hydrogels with varying Fe^3+^ amounts. (**b**) Tensile stress–strain curves of hydrogels with varying AM:SA ratios. (**c**) Tensile stress–strain curves of hydrogels with varying crosslinker amounts. (**d**) Young’s modulus, tensile strength, toughness, and strain at break of hydrogels with varying Fe^3+^ amounts. (**e**) Young’s modulus, tensile strength, toughness, and strain at break of hydrogels with varying AM:SA ratios. (**f**) Young’s modulus, tensile strength, toughness, and strain at break of hydrogels with varying crosslinker amounts. (**g**) P1M0.5Fe1 hydrogels before and after stretching at over 2700% strain.

In this study, tensile tests were performed on the as-prepared hydrogel samples. The corresponding stress–strain curves are shown in Figure 2a–c, representing the variations of x, y, z value, respectively. Quantitative mechanical properties, e.g., Young’s modulus, toughness, strain at break, and tensile strength, were calculated from the stress–strain curves plotted in Figure 2d–f. Figure 2g is a photograph documenting the tensile testing of a P1M0.5Fe1 specimen, which exhibited an exceptional stretchability with a strain at break of over 2500%.

According to Figure 2a,d, the P1M0.5Fe0 sample displayed a characteristic ductile behavior, with a strain at break of ~900%, a tensile strength of 155 kPa, and a Young’s modulus of 100 kPa. The addition of Fe^3+^ softened the PAM-SA hydrogel. As evidenced in Figure 2a,d, the P1M0.5Fe1 sample demonstrated a reduced Young’s modulus at 50 kPa, verifying the significant softening. Meanwhile, the strain at break also increased drastically to ~2500%, indicating an ultra-stretchable property. In addition, the toughness was also substantially increased from 0.9 MJ/m^3^ to 2.3 MJ/m^3^, reflecting a high work of fracture. The simultaneous softening–toughening behavior potential originated from Fe^3+^ participation in the polymerization kinetics. Pan et al. [27] reported that the Fe^3+^ underwent monomer-mediated photoreduction under visible light, generating Fe^3+^/Fe^2+^ redox pairs that initiated free radical polymerization. Zhang et al. [28] demonstrated that these pairs participated in RAFT polymerization, augmenting radical generation. The resulting increased radical concentration shortened polymer chains and therefore reduced the Young’s modulus and softened the hydrogels. (Further investigation on the mechanism are underway.) On the other hand, the Fe^3+^- COO^−^ chelation established reversible physical crosslinks, contributing to the high toughness through the energy dissipation mechanism. Beyond the optimal Fe^3+^ concentration, mechanical properties declined. P1M0.5Fe2 sample maintained a similar Young’s modulus and a stress–strain curve below the 1500% strain region, while the strain at break was decreased to ~1800%. P1M0.5Fe3 showed an additional influence on the strain at break and Young’s modulus (green curves, Figure 2a,d), reduced to 900% and 10 kPa, respectively.

The mass ratio of AM:SA was also an important parameter for tuning the mechanical properties. As shown in Figure 2b,e, increasing P to 2 and 3 significantly increased the hydrogel stiffness, as their Young’s moduli rose to 100 kPa and 150 kPa, respectively. However, this stiffening coincided with reduced strain at break (1250% and 1000%, respectively). This behavior likely originated from more compact polymer networks at higher AM content, where enhanced intermolecular hydrogen bonding dominated over the repulsion of SA repeating units, which diminished the intermolecular interactions [26]. Conversely, decreasing P produced a softening trend. P0.5M0.5Fe1 samples exhibited a lower Young’s modulus of 50 kPa but a much higher strain at break of 2750%, demonstrating a soft but highly stretchable nature. The toughness also achieved the highest value of 2.4 MJ/m^3^ for this composition, suggesting optimal synergy between Fe^3+^ chelation and AM-SA hydrogen bonding. However, at P = 0.3 (P0.3M0.5Fe1), tensile strength, Young’s modulus, and strain at break were all reduced, indicating Fe^3+^ ion aggregation that emphasizes stress concentration.

The mechanical properties of the hydrogel were further modulated by varying the amount of crosslinkers (M value). As shown in Figure 2c,f, when increasing the M value to 3, Young’s modulus increased to 100 kPa. Simultaneously, the strain at break was reduced to 900%. These trends align with established crosslinking principles where sparse networks produce soft and tough hydrogels.

To assess the long-term mechanical stability, hydrogels were stored in sealed bags for 1 month prior to tensile testing. Post-storage analysis revealed reductions in both strain at break and tensile strength across all formulations (Figure S3). Importantly, retained extensibility remained substantial (>1000% strain), confirming enduring stretchability. Concurrently, increased curve slopes indicated elevated Young’s moduli—consistent with hydrogel stiffening from dehydration during storage, which diminishes polymer chain mobility and increases effective crosslink density [29].

While recent literature reports one-pot hydrogel synthesis on a multivalent ion-based hydrogel (Table S1), our strategy achieves exceptional tunability, spanning unprecedented strain at break (>2500%) and Young’s modulus (10–170 kPa) ranges while maintaining recorded toughness up to 2.4 MJ/m^3^. This combination of extreme extensibility, wide modulus adjustability, and fracture resistance represents a significant advancement in high-performance hydrogel design.

### 2.3. Compressive Tests

**Figure 3:**
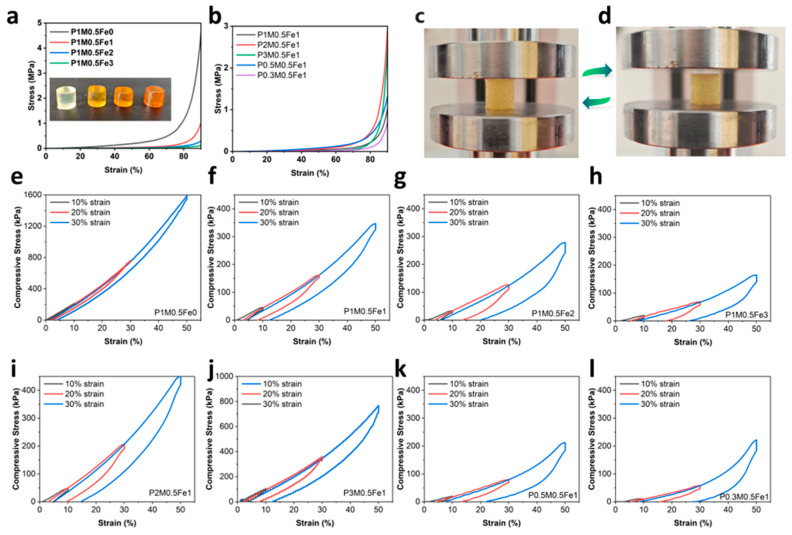
(**a**) Compressive stress–strain curves of hydrogels with varying Fe^3+^ amounts. Inset: compressive test samples with different Fe^3+^ amounts. (**b**) Compressive stress–strain curves of hydrogels with varying AM:SA ratios. (**c**) P1M0.5Fe1 sample before compressing to 90% strain. (**d**) P1M0.5Fe1 sample after compressing to 90% strain. (**e**–**h**) Compressive load–unload cycles with samples with varying Fe^3+^ amounts. (**i**–**l**) Compressive load–unload cycles with samples varying AM:SA ratios.

To verify the trend observed in tensile tests, complementary compressive tests were performed. The photograph of samples with different Fe^3+^ amounts is shown in Figure 3a inset, where a higher Fe^3+^ concentration introduced a darker brown color. Figure 3a revealed a systematic decrease in compressive strength at 90% strain from 5 MPa to 0.2 MPa with an increasing Fe^3+^ amount, confirming the composition-dependent softening. Monomer composition variations similarly followed the trend of tensile tests. P2M0.5Fe1 and P3M0.5Fe1 exhibited a higher compressive strength than the P1M0.5Fe1 sample, while P0.3M0.5Fe1 and P0.5M0.5Fe1 showed a reduced compressive strength, as shown in Figure 3b. Notably, despite softening, the P1M0.5Fe1 specimen demonstrated exceptional recovery after 90% compression (Figure 3c,d). Figure S4 is a photograph documenting the hydrogels before and after compression to 90% strain. All compositions exhibited complete shape recovery except for the P0.3M0.5Fe1 sample, which displayed permanent deformation after the compression.

### 2.4. Cyclic Load–Unload Tests

In order to evaluate fatigue-resistance properties, cyclic compressive load–unload cyclic tests were performed at varying strains (10%, 20%, 30%) across different compositions. As shown in Figure 3e and Figure S5, P1M0.5Fe0 demonstrated a high peak strength of 225 kPa (10% strain), 806kPa (30% strain), and 1607 kPa (50% strain). In the 10% strain cycle, P1M0.5Fe0 exhibited a near-perfect elasticity (overlap between the compress and release curves), yielding minimal dissipated energy of 0.012 MJ/m^3^. Increasing the strain to 30% and 50% elevated the dissipated energy to 0.12 and 0.43 MJ/m^3^, as shown in Figure S6. In contrast, P1M0.5Fe1 sample displayed a pronounced hysteresis loop, with dissipated energy of 0.017, 0.095, and 0.26 MJ/m^3^ at 10%, 30%, and 50% strain, while the peak strengths were 39 (10% strain), 160 (30% strain), and 348 kPa (50% strain) (Figure 3f, Figures S5 and S6). P1M0.5Fe2 demonstrated analogous behavior, in which the enhanced energy dissipations were calculated to be 0.020, 0.13, and 0.34 MJ/m^3^. P1M0.5Fe3 showed a comparable hysteresis loop but reduced peak strength. Importantly, Fe^3+^-incorporated hydrogels achieved a comparable level of dissipated energy to P1M0.5Fe0 at significantly reduced stiffness. To better understand the energy dissipation properties, we calculated the dissipation efficiency as the percentage ratio of the hysteresis loop area to total input energy (area under the loading curve), as illustrated in Figure S7. P1M0.5Fe0 only dissipated 10% of energy, while the samples with Fe^3+^ achieved up to 90% efficiency. With the increase in Fe^3+^ concentration, the dissipation efficiency increased, implying enhanced energy dissipation capacity at higher physical crosslinking density. Additionally, the consecutive curves showed progressive overlap with each other, indicating outstanding resilience to prevent permanent deformation. Analogous recovery was observed in tensile cyclic tests (Figure S8a–h). At fixed Fe^3+^ concentration, the AM:SA ratio tuned the compressive strength while preserving the energy dissipation capability. As shown in Figure 3i–l, increased SA content reduced compressive modulus but raised dissipation efficiency. This inverse strength–dissipation relationship directly implicates Fe^3+^-COO- coordination as the dominant energy dissipation pathway.

### 2.5. Degree of Swelling

**Figure 4:**
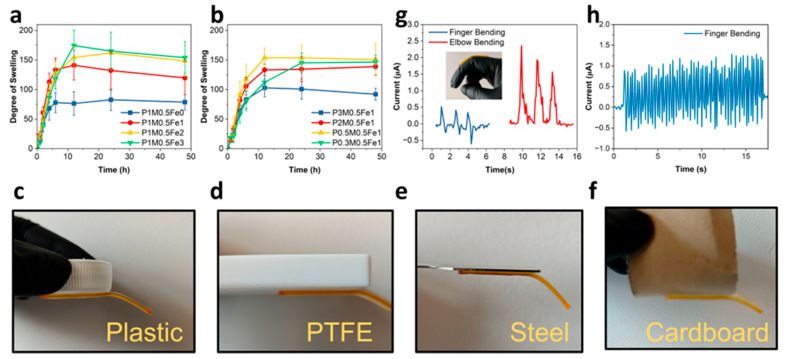
(**a**) Degree of swelling of hydrogels with varying Fe^3+^ amounts. (**b**) Degree of swelling study of hydrogels with varying AM:SA ratios. (**c**–**f**) P1M0.5Fe1 samples adhere to different material surfaces. (**g**) P1M0.5Fe1 electrical signal for sensing finger and elbow bending movements. (**h**) Continuous finger bending for multiple cycles of testing.

Hydrogel swelling capacity critically governs performance in key applications, including drug delivery, wound dressing, and contaminant absorbing [30]. To analyze the swelling behavior of different compositions, hydrogel samples were immersed in deionized (DI) water at room temperature. And the hydrogels were taken out at desired time intervals, surface-dried with paper tissue, and weighed to determine the mass change. The degree of swelling (S) was then calculated with the equation $S=(W_{t}-W_{0})/W_{0}$, where $W_{t}$ is the swollen weight at time t and $W_{0}$ is the initial weight. The results are shown in Figure 4a,b. P1M0.5Fe0 demonstrated the lowest equilibrium degree of swelling, while with the increase of Fe^3+^, the degree of swelling gradually increased and the swelling speed (slope of the swelling curves) also increased. With the increase in SA amount, the degree of swelling also increased. These could be attributed to the polymer chain length difference during polymerization under different Fe^3+^ concentrations, as the high Fe^3+^ concentration hindered the polymerization.

### 2.6. Self-Adhesive, Ionic Conductivity, and Sensory Applications

As demonstrated in Figure 4c–f, the hydrogel exhibited universal adhesion to diverse substrates, including plastics, PTFE, metals, and cardboard. The obtained self-adhesive behavior was possibly mediated by COO^−^ groups. To verify, we prepared a SA-free polyacrylamide hydrogel (homo-PAM) as a control. Movie S1 confirms that the homo-PAM hydrogel failed to adhere to steel, whereas Movie S2 shows that the P1M0.5Fe1 adhered to the steel surface and required mechanical peeling for detachment. This proves that the COO^−^ groups enabled adhesion, consistent with previous publications [31].

Ionic conductivity is essential for hydrogel-based strain sensors as it governs the signal transduction efficiency and sensitivity. As indicated in Figure S9, ionic conductivity can be adjusted by increasing Fe^3+^ from 0.22 S m^−1^ (P1M0.5Fe0) to 0.25 S m^−1^ for P1M0.5Fe3, aligning with previous publications [26]. Notably, after 1 month of storage in a sealed bag (Ziplock bag), the sample maintained >96% while retaining the Fe^3+^-dependent trend. Taking P1M0.5Fe3 as an example, the conductivity decreased from 0.25 S m^−1^ to 0.24 S m^−1^. P2M0.5Fe1 demonstrated a higher conductivity as compared to other hydrogel samples of modifying AM:SA ratios. Conversely, a high SA ratio reduced the conductivity, likely due to the elevated polyelectrolyte chain causing electrostatic blobs to overlap. This phenomenon constricts the ion transport pathway and reduces the counterion mobility [32].

Previous results showed that the PxMyFez hydrogels offered tunable softness, conductivity, adhesion, and stretchability, demonstrating strong potential for sensory applications. To demonstrate its functionality, we mounted the hydrogel to both the finger and the elbow joint and recorded distinct real-time electrical signals. As shown in Figure 4g, both joint motions generated unique signal profiles with rapid response/recovery time (around 90 ms), comparable with high-performance ionic sensors in the literature [33,34]. Although Figure 2 and Figure 3 indicate that strain hardening occurred at high strains (e.g., >500% in P1M0.5Fe1, beyond typical sensor operational ranges), Figure 3e,f and Figure S6 confirm its absence within the functional sensing window (<50% compressive strain; <300% tensile strain). Cyclic finger bending tests maintained consistent electrical signal output (Figure 4h), indicating exceptional durability under working conditions. Halving precursor concentration enhanced response currents (Figure S10), highlighting signal adaptability.

Gauge factors (GFs) were characterized using a universal testing machine equipped with a multimeter. As shown in Figure S11, the GF of the P1M0.5Fe0 showed the lowest value, as the free ion concentrations were low. P1M0.5Fe1, P1M0.5Fe2, and P1M0.5Fe3 showed similar slopes, indicating minimal Fe^3+^ concentration dependence. Interestingly, P3M0.5Fe1 showed the highest slope throughout the whole 0–1000% strain region, suggesting that AM:SA ratio optimization improves sensitivity. Conversely, increased SA content reduced GF slopes—aligning with ionic conductivity trends—attributed to restricted ion mobility from polyelectrolyte chain overlap.

## 3. Conclusions

In summary, this work established a novel synthesis strategy of soft, adhesive, and ultra-stretchable AM-SA copolymer hydrogels. The hydrogel was prepared in the presence of Fe^3+^ ions via PET-RAFT polymerization. This preparation is oxygen-tolerant and requires no external heat, enabling a scalable route for Fe^3+^-incorporated hydrogels. By adjusting the Fe^3+^ concentration, the modulation of mechanical properties has been achieved: Young’s modulus (10 to 150 kPa), toughness (0.26 to 2.3 MJ/m^3^), and strain at break (800% to 2500%). The hydrogels also exhibit excellent compressibility (90% strain recovery), energy dissipation (>90% dissipation efficiency), and universal adhesion to diverse surfaces. We have also demonstrated the ability to adjust the degree of swelling via modulating the Fe^3+^ concentration and AM:SA feed ratios. In addition, the hydrogels showed a self-adhesive property on multiple surfaces. Finally, Fe^3+^-incorporated hydrogels demonstrated high effectiveness as strain sensors for monitoring finger/elbow movements, with gauge factors dependent on composition. This study delivers an advanced strategy in hydrogel manufacturing: ambient, scalable PET-RAFT polymerization of Fe^3+^-coordination networks with tunable mechanics for next-generation wearable devices.

## 4. Materials and Methods

### 4.1. Materials

Sodium acrylate (SA), Acylamide (AM), N,N’-Methylenebisacrylamide (MBAA), Eosin Y (EY), Triethanolamine (TEOH) and 4-Cyano-4-(((dodecylthio)carbonothioyl)thio)pentanoic acid (CDTPA) and Ferric Chloride (FeCl_3_) were purchased from Shanghai Aladdin Biochemical Technology Co., Ltd. (Shanghai, China). Deionized (DI) water (resistivity > 18.2 MΩ cm) was used for all the samples.

### 4.2. Fe^3+^ Modulated In Situ Formation of PAM-SA Hydrogels

In situ preparation of Fe^3+^ modulated hydrogel was performed as follows. Firstly, EY, TEOH, and CDTPA were dissolved in water to prepare a concentrated solution 1. MBAA and FeCl_3_ were dissolved in water to prepare a 10 mg/mL solution. The desired amount of SA and AM was added to a 20 mL vial and dissolved in water, followed by the adding of 50 μL MBAA solution and 50 μL of concentrated solution 1. After vortex mixing, the desired amount of FeCl_3_ solution was added and vortex-mixed for 30 s. The final product was then sonicated for 1 min to remove bubbles. Then, the final solution was added to a PTFE or silicone mold for PET-RAFT polymerization for the corresponding tests. The mold was placed under a parallel LED light source (530 nm, 2.79 mW/cm^2^) for 1 h. At least 3 samples were further tested with mechanical and electrical tests. The calculated mechanical properties and ion conductivity value were given as the average of 3 samples. The FTIR spectra were measured with a Thermo Fisher Nicolet IS50 (Waltham, MA, USA).

### 4.3. Tensile Tests

Tensile tests were conducted with an HZ-1004B electronic universal test machine (Dongguan Lixian Experimental Instrument, Dongguan, China). The tensile tests were conducted on a rectangular specimen designed as 20 mm wide × 50 mm long × 2 mm thick, prepared with the corresponding PTFE mold. The deformation speed was set as 100 mm/min unless otherwise mentioned. Tensile stress was calculated as the quotient of the load force divided by the original cross-sectional area. The tensile strain was calculated by dividing the displacement by the original length. The toughness of the hydrogel was calculated by the area under the strain–stress curve.

### 4.4. Compressive Tests

The compressive specimen was a 10 mm diameter × 10 mm height cylinder, prepared with a silicone mold. The compressive stress was calculated by dividing the load force by the original area. The deformation speed was set as 10 mm/min unless otherwise mentioned, and the stop strain was set as 90% strain.

### 4.5. Load–Unload Cycles

The compressive load–unload cyclic tests were performed using the cylinder samples with a continuous compress and release of strain at 10%, 30%, and 50%. The dissipated energy of the hydrogel was calculated by the area between the load–unload curves. The percentage of dissipated energy was calculated as the ratio of the area between the load–unload curves divided by the area under the load curves.

### 4.6. Swelling Tests

The swelling tests were performed by immersing the as-prepared sample in DI water. The samples were weighted before immersing and were taken out at the desired time, wiped with paper tissue to remove excessive moisture, and weighted. The degree of swelling (S) was calculated with the equation $S=(W_{t}-W_{0})/W_{0}$, where $W_{t}$ is the swollen weight at time t and $W_{0}$ is the initial weight.

### 4.7. Sensing Finger and Elbow Bending

The sensing of finger and elbow motions was performed by using an electrochemical workstation (HY-Cube, Ichy, Shenzheng, China). The hydrogel was adhered to the finger and the elbow to sense the bending behavior.

### 4.8. Ionic Conductivity

The ionic conductivity was measured with a four-probe resistance mode with Keitheley 2000 Multimeter (Tektronix, Inc., Beaverton, OR, USA). The conductivity was calculated by the equation σ=L/RA, where σ was the ionic conductivity, L was the length of the sample, A was the cross-sectional area of the sample, and R represented the calculated resistance of the sample. The reported conductivity value was an average of three different samples.

### 4.9. Gauge Factor (GF) Tests

GF was measured with the universal test machine connected with a Keitheley 2000 Multimeter (Tektronix, Inc., Beaverton, OR, USA). The tensile speed was set as 200 mm/min. The changes in the relative resistance of the hydrogel were defined by the following equation $\frac{∆R}{R_{0}}\times 1$00%, where ∆R represented the change of electrical resistance during stretching, and $R_{0}$ was the initial electrical resistance. GF was then calculated using the following equation $GF=\frac{∆R}{R_{0}\varepsilon }$, where ε is the strain of the sample.

## Figures and Tables

**Table 1:** Final compositions of different hydrogel samples.

Sample (PxMyFez)	AM (g)	SA (g)	MBAA (mg)	FeCl_3_ (mg)	EY (mg)	TEOH (mg)	CDTPA (mg)	Water (mL)
P1M0.5Fe0	0.50	0.50	0.5	0	0.05	2	0.25	1
P1M0.5Fe1	0.50	0.50	0.5	1	0.05	2	0.25	1
P1M0.5Fe2	0.50	0.50	0.5	2	0.05	2	0.25	1
P1M0.5Fe3	0.50	0.50	0.5	3	0.05	2	0.25	1
P0.3M0.5Fe1	0.25	0.75	0.5	1	0.05	2	0.25	1
P0.5M0.5Fe1	0.33	0.67	0.5	1	0.05	2	0.25	1
P2M0.5Fe1	0.67	0.33	0.5	1	0.05	2	0.25	1
P3M0.5Fe1	0.75	0.25	0.5	1	0.05	2	0.25	1
P1M1Fe1	0.50	0.50	1	1	0.05	2	0.25	1
P1M2Fe1	0.50	0.50	2	1	0.05	2	0.25	1
P1M3Fe1	0.50	0.50	3	1	0.05	2	0.25	1
*p* value (x):	Adjusting AM:SA ratio							
M value (y):	Adjusting crosslinker (MBAA) amount							
Fe value (z):	Adjusting Fe^3+^ amount							

## Data Availability

The original contributions presented in this study are included in the article. Further inquiries can be directed to the corresponding author.

## Supplementary Materials

The following supporting information can be downloaded at: https://www.mdpi.com/article/10.3390/gels11080586/s1, Figure S1: P1M0.5Fe0-P1M0.5Fe3 precursor solution stored in dark for 1 h and endured subsequent irradiation for 1 h. Figure S2: FTIR spectra of P1M0.5Fe0-P1M0.5Fe3 hydrogels and zoomed in area from 1720 to 1620 cm^−1^. Figure S3: Tensile tests for hydrogel samples stored for 1 month. Figure S4: Hydrogel samples before and after compression of 90% strain. Figure S5: Peak strengths Obtained from Compressive Load-Unload Cycle Tests. Figure S6: Dissipated Energy Calculated from Compressive Load-Unload Cycle Tests. Figure S7: Percent of Dissipated Energy Calculated from Compressive Load-Unload Cycle Tests. Figure S8: Tensile Load-Unload Cycle Tests for different hydrogel samples. Figure S9: Ionic conductivity of as prepared hydrogel samples and samples stored for 1 month in a sealed bag. Figure S10: Finger bending and elbow bending signal using hydrogel sample with half of the concentration. Figure S11: Gauge Factor tests for the hydrogel samples. Table S1: Table of mechanical properties of recent published work on multi-valent ion incorporated hydrogels [22,23,34,35,36,37,38,39]. Movie S1: Peel homo-PAM from steel surface. Movie S2: Peel P1M0.5Fe1 from steel surface.
