# Supplementary material for: Fe3+-Modulated In Situ Formation of Hydrogels with Tunable Mechanical Properties
Source: Gels. 2025 Jul 30;11(8):586. doi: 10.3390/gels11080586 (PMC12385994; doi:10.3390/gels11080586)
Supplement: Supplementary file 1 [file gels-11-00586-s001.zip › Gels SI.pdf]

Supporting Information

Fe^{3+} modulated in-situ formation of hydrogels with tunable mechanical properties

Lihan Rong [1,2,*], Tianqi Guan [1], Xinyi Fan [1] Wenjie Zhi [1], Rui Zhou [1], Feng Li [1] and Yuyan Liu [1]

1 College of Physics and Electronic Information Engineering , Neijiang Normal University, Neijiang 641112, P.R. China
2 Neijiang Optoelectronic Devices Engineering Research Center, Neijiang 641112, P.R. China
* Correspondence: rlihan@163.com

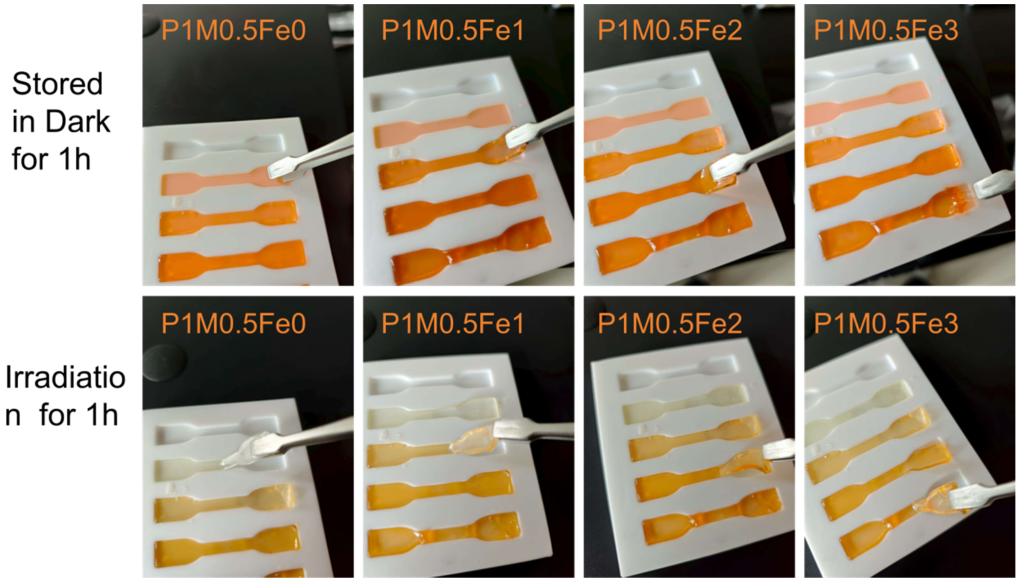

Figure S1. P1M0.5Fe0-P1M0.5Fe3 precursor solution stored in dark for 1 h and endured subsequent irradiation for 1 h.

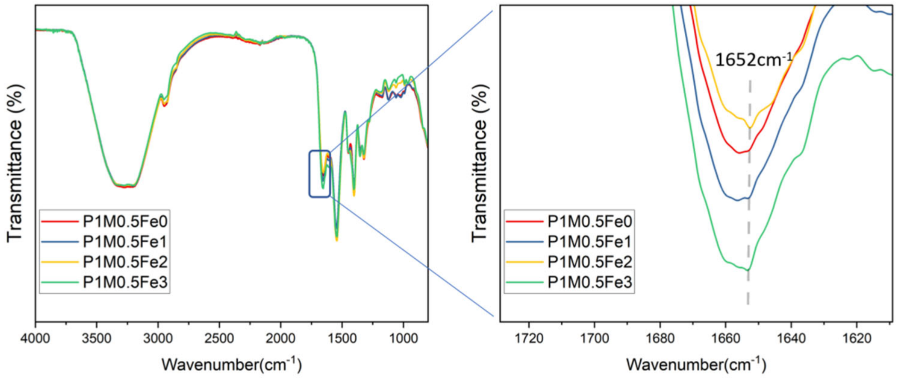

Figure S2. FTIR spectra of P1M0.5Fe0-P1M0.5Fe3 hydrogels and zoomed in area from 1720 to 1620 cm^{-1}.

Table S1. Table of mechanical properties of recent published work on multi-valent ion incorporated hydrogels.

Sample	Preparation method	Strain at break (%)	Toughness (MJ/m^3)	Young's modulus (kPa)
lignosulfonate sodium (LS)-doped polyacrylic acid(PAA)[1]	Two step process: polymeriztion +immersing in Fe^{3+} solution	500~3000	0.1~0.25	20~50
bacterial cellulose nanowhisker (BCW),tannic acid(TA), PAA, Fe^{3+} and glycerol/water[2]	One pot synthesis with Fe^{3+}	1250~2250	\	\
Lignosulfonate, poly(ethylene glycol) diglycidyl ether (PEGDGE)[3]	Two step process: polymeriztion +immersing in Fe^{3+} solution	50	\	\
Polyaniline(PANI), Poly(acrylic acid-co-acrylamide (PAA-co-PAM)[4]	Three step process: polymeriztion +immersing in Fe^{3+} solution +immersing in PANI solution	150~900	\	7-69
Polyvinyl alcohol (PVA)/gelation Fe^{3+}[5]	Two step process: polymeriztion +immersing in Fe^{3+} solution	300~837	\	30~130
copolymer of AA and hydroxyethyl methacrylate (HEMA) Carboxy methyl cellulose (CMC), polydopamine (PDA) and Fe^{3+}[6]	One pot synthesis with Fe^{3+}	143~1175	0.5~1.3	\
PAA, PVA and Fe^{3+}[7]	One pot synthesis with Fe^{3+}	850~1300	\	100~150
PAM/Copper Alginate [8]	Two step process: polymeriztion +immersing in Cu^{2+} solution	1500-2500	\	\
Zn^{2+}/cellulose/PAA[9]	Two step process: Zn functionalizetion+polymeriztion	440~620	0.09-0.18	\
This work	One pot synthesis with Fe^{3+}	900~2750	0.2~2.4	10~170

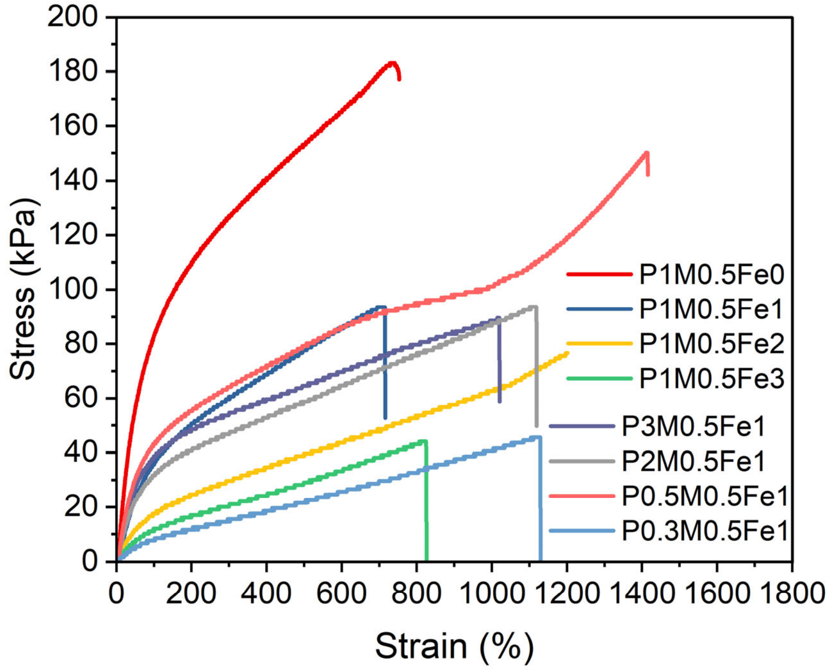

Figure S3. Tensile tests for hydrogel samples stored for 1 month.

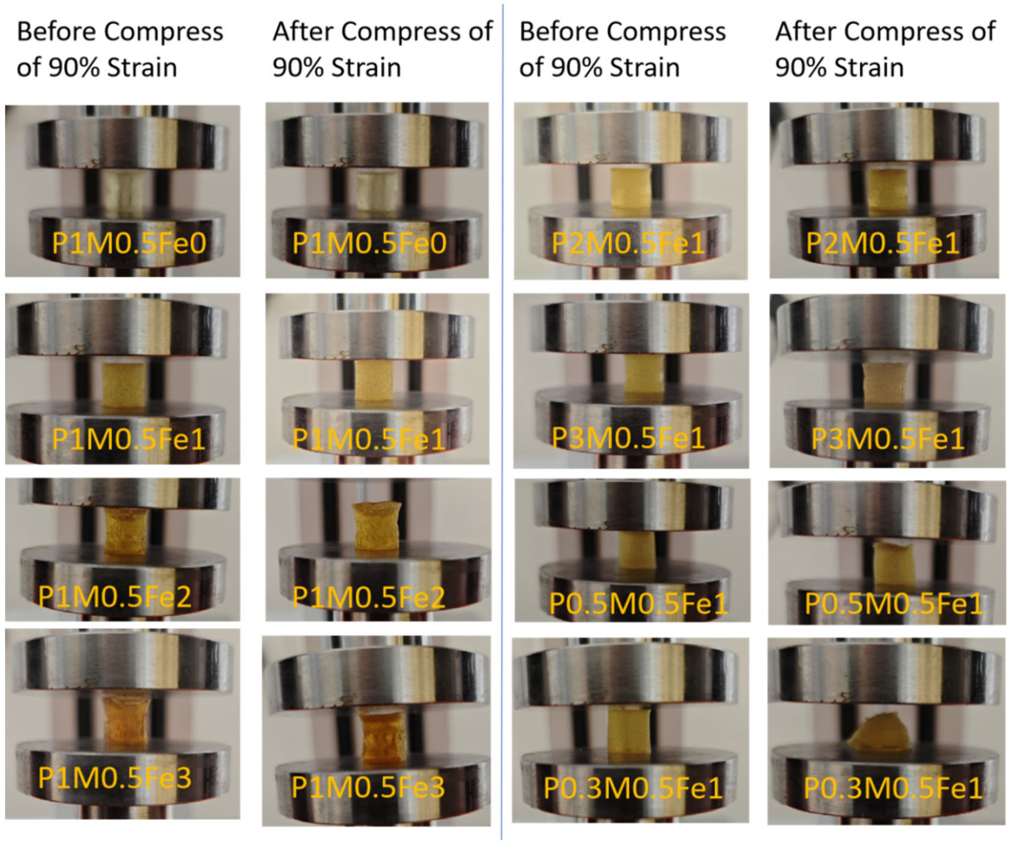

Figure S4. Hydrogel samples before and after compression of 90% strain

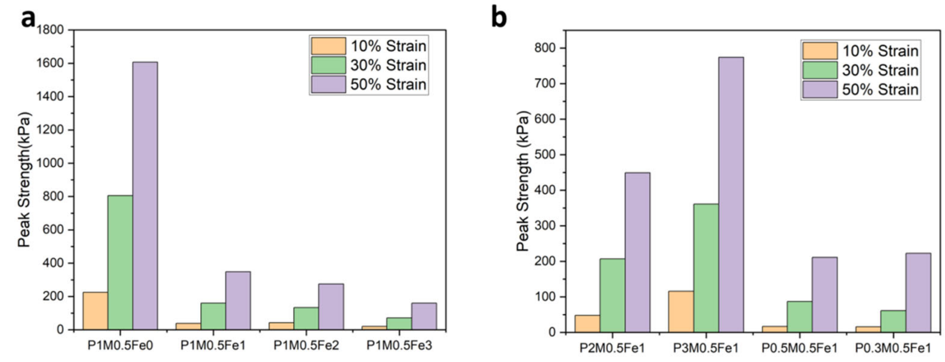

Figure S5. Peak strengths Obtained from Compressive Load-Unload Cycle Tests

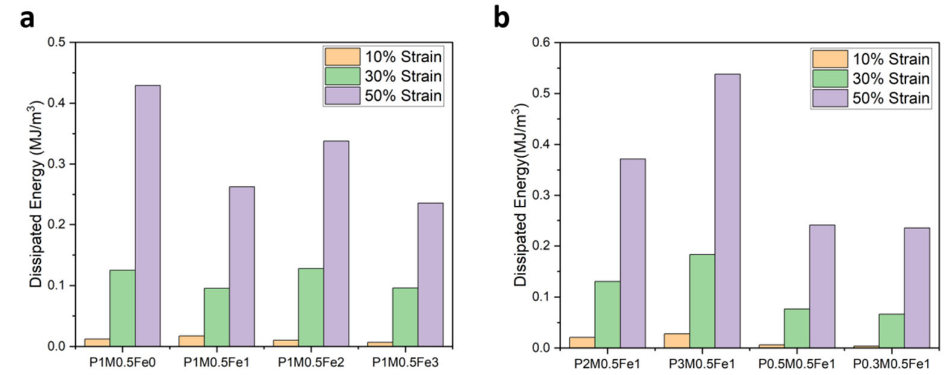

Figure S6. Dissipated Energy Calculated from Compressive Load-Unload Cycle Tests

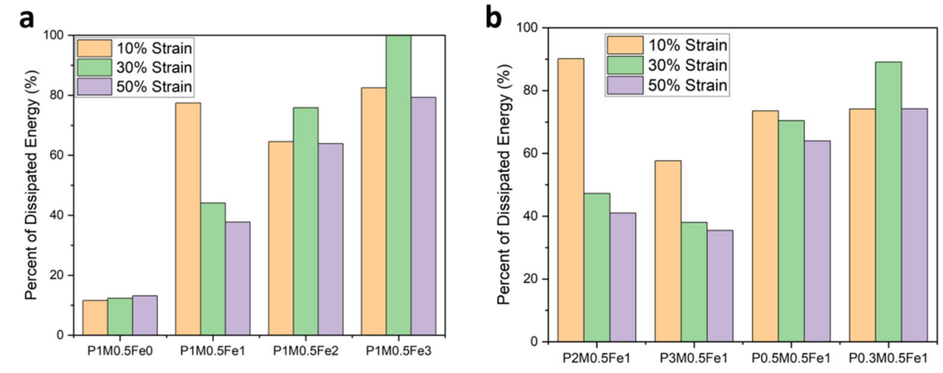

Figure S7. Percent of Dissipated Energy Calculated from Compressive Load-Unload Cycle Tests

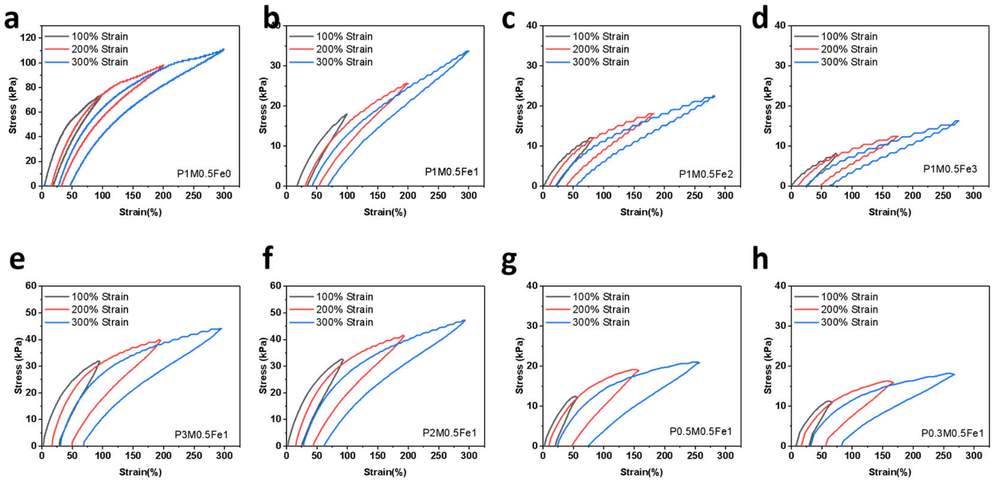

Figure S8. Tensile Load-Unload Cycle Tests for different hydrogel samples

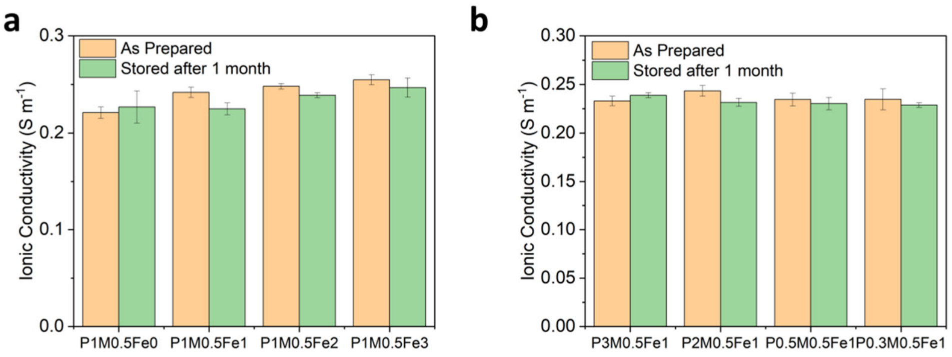

Figure S9. Ionic conductivity of as prepared hydrogel samples and samples stored for 1 month in a sealed bag.

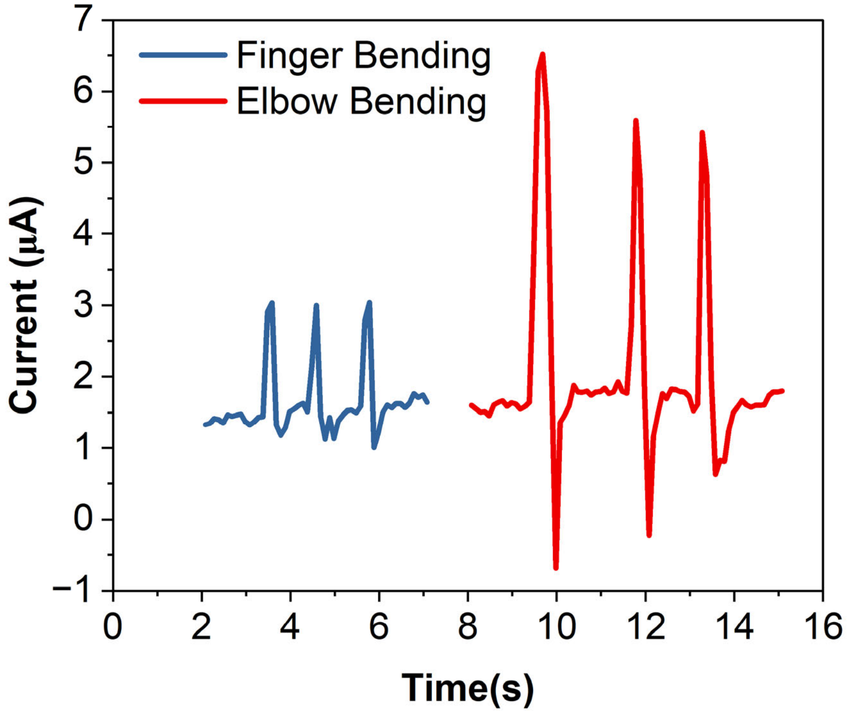

Figure S10. Finger bending and elbow bending signal using hydrogel sample with half of the concentration.

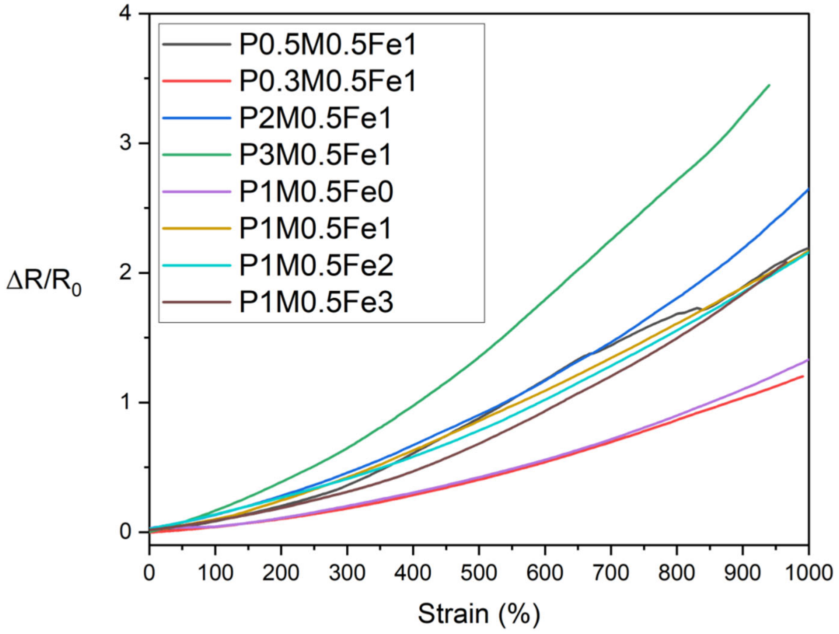

Figure S11. Gauge Factor tests for the hydrogel samples

Reference

1. Wang, Q., et al., *An oriented Fe3+-regulated lignin-based hydrogel with desired softness, conductivity, stretchability, and asymmetric adhesiveness towards anti-interference pressure sensors.* International Journal of Biological Macromolecules, 2021. **184**: p. 282-288.
2. Wang, J., et al., *Tannic acid-Fe3+ activated rapid polymerization of ionic conductive hydrogels with high mechanical properties, self-healing, and self-adhesion for flexible wearable sensors.* Composites Science and Technology, 2022. **221**: p. 109345.
3. Mondal, A.K., et al., *Design of Fe3+-Rich, High-Conductivity Lignin Hydrogels for Supercapacitor and Sensor Applications.* Biomacromolecules, 2022. **23**(3): p. 766-778.
4. Sun, X., et al., *Fe3+-Coordination mediated synergistic dual-network conductive hydrogel as a sensitive and highly-stretchable strain sensor with adjustable mechanical properties.* Journal of Materials Chemistry B, 2022. **10**(9): p. 1442-1452.
5. Wang, Y., et al., *Highly sensitive and multifunctional Fe3+ enhanced PVA/gelatin multi-network hydrogels with wide temperature range environmental stability for wearable sensors.* International Journal of Biological Macromolecules, 2025. **311**: p. 143606.
6. Wang, J., et al., *A highly stretchable and self-adhesive cellulose complex hydrogels based on PDA@Fe3+ mediated redox reaction for strain sensor.* International Journal of Biological Macromolecules, 2024. **281**: p. 136307.
7. Wang, D., et al., *A Conductive Hydrogel Based on GaIn and PVA/PAA/Fe3+ for Strain Sensor and Physiological Signal Detection.* ACS Applied Polymer Materials, 2021. **3**(10): p. 5268-5276.
8. Zhang, Z., et al., *Polyacrylamide/Copper-Alginate Double Network Hydrogel Electrolyte with Excellent Mechanical Properties and Strain-Sensitivity.* Macromolecular Bioscience, 2022. **22**(2): p. 2100361.
9. Wang, Y., et al., *Zinc-ion engineered Plant-based multifunctional hydrogels for flexible wearable strain Sensors, Bio-electrodes and Zinc-ion hybrid capacitors.* Chemical Engineering Journal, 2023. **465**: p. 142917.
